# Activity of *α*_1_-Antitrypsin and Some Lysosomal Enzymes in the Blood Serum of Patients with Chronic Obstructive Pulmonary Disease after Smoking Cessation

**DOI:** 10.1155/2015/176582

**Published:** 2015-01-31

**Authors:** Bartosz Woźniak, Alina Woźniak, Jacek Konca, Dariusz Górecki, Celestyna Mila-Kierzenkowska, Michał Szpinda, Paweł Sutkowy, Roland Wesołowski

**Affiliations:** ^1^Department of Neurosurgery, Stanisław Staszic Specialist Hospital, Rydygiera 1, 64-920 Piła, Poland; ^2^The Chair of Medical Biology, Collegium Medicum of Nicolaus Copernicus University, Karłowicza 24, 85-092 Bydgoszcz, Poland; ^3^Dental Medical Clinic Demeter, Białostocka 7, 03-741 Warszawa, Poland; ^4^Family Medicine Center, Przesmyk 2/4, 87-100 Toruń, Poland; ^5^Department of Normal Anatomy, Collegium Medicum of Nicolaus Copernicus University, Łukasiewicza 1, 85-801 Bydgoszcz, Poland

## Abstract

The activity of *α*_1_-antitrypsin (AAT) and the lysosomal enzymes, cathepsin D (CTS D), arylsulfatase (ASA), and acid phosphatase, (AcP) was determined in patients with COPD (GOLD category A). Moreover, the diagnostic usefulness of these parameters in blood serum was assessed along with establishing whether smoking cessation affects these parameters. The study included 70 patients with COPD who ceased smoking (study group) and two control groups of 33 subjects each: nonsmokers without COPD (control I) and patients with COPD who continued smoking (control II). In control I, blood was taken once and in control II, at the start of the experiment and after the 1st, 2nd, and 3rd months. AAT in the patients exhibited higher activity than in the healthy subjects at all time points. AAT activity in the patients before the start of the experiment was ~80% higher (*P* < 0.001) than in control I. No statistically significant differences in CTS D, ASA, and AcP activity were found. COPD involves increased AAT activity and unchanged activities of the assessed lysosomal enzymes. Three-month tobacco abstinence does not affect these parameters in peripheral blood. Determining the AAT levels in blood serum can be used in the diagnostics of COPD.

## 1. Introduction

Chronic obstructive pulmonary disease (COPD) is the second (after lung cancer) cause of death due to respiratory diseases in Europe [1]. It is characterized by a limited air flow through the airways. Ventilation disturbances in COPD patients are caused by airway obstruction resulting from a chronic inflammatory process in the bronchi [2]. One of the factors leading to the development of chronic inflammation in the airways is cigarette smoking [3]. The primary role in the inflammatory process in COPD is played by macrophages whose number significantly increases in the airways, lung parenchyma, bronchoalveolar lavage (BAL), and sputum and correlates with the severity of the disease [4]. COPD is accompanied by changes affecting not only the lungs. The persistent inflammation in the lungs stimulates the release of proinflammatory cytokines and chemokines into the circulating blood. These factors stimulate the liver, adipose tissue, and bone marrow to release large amounts of leukocytes, C-reactive protein (CRP), interleukins 6 and 8 (IL-6 and IL-8), fibrinogen, and tumor necrosis factor-*α* (TNF-*α*). As a result, these processes lead to a low-grade systemic inflammatory process [5, 6].

In the pathogenesis of COPD, apart from the inflammation, an important role is played by two other processes: oxidative stress [7] and imbalance in the activity of proteases and antiproteases in the lung parenchyma [8]. The main sources of proteases in the lungs are macrophages and neutrophils. Among the proteases proven as important in COPD course are neutrophil elastase, matrix metalloproteinases (MMP-2, MMP-9, and MMP-12, in macrophages), and cathepsins S, L (in macrophages), and G, as well as proteinase-3 (in neutrophils) [6]. The increased activity of proteolytic enzymes in COPD leads to the destruction of alveolar walls and, consequently, to lung emphysema. Neutrophil elastase constitutes the primary elastolytic mechanism in patients with *α*_1_-antitrypsin (AAT) deficiency, while in patients with COPD associated with tobacco smoking, a more important role is played by cathepsins and matrix metalloproteinases [9].

AAT is a protein belonging to serine protease inhibitors. It is synthesized mainly in the liver and belongs to the acute phase plasma proteins associated with acute inflammatory episodes including infectious and obstructive lung diseases [10]. It is hypothesized that plasma AAT may be a noninvasive marker of smoking-related inflammation or COPD [10]. Among the cell organelles playing the key role at many stages of the inflammatory process are lysosomes [11]. Cathepsin D (CTS D) is the best-characterized aspartic protease occurring in lysosomes. Some reports indicate a functional role of this enzyme in lung diseases [12]; however, there are no data in the literature regarding the changes in the activity of CTS D in COPD, especially that determined in blood serum. Among other lysosomal enzymes whose activity in blood serum was found to change in various diseases are acid phosphatase (AcP) and arylsulfatase (ASA) [13, 14].

The aim of the study was to determine the activity of the inhibitor of proteases, AAT, CTS D, ASA, and AcP in patients with COPD. Moreover, the usefulness of determining these parameters in blood serum in the diagnostics of COPD was assessed and an attempt to establish whether smoking cessation for three months may induce changes in the activity of the measured lysosomal enzymes and AAT was made.

## 2. Materials and Methods

The study involved 70 patients with COPD, aged 26 to 72 (mean age = 48.8 ± 12.1 years), treated at the Specialist Family Medicine Center in Toruń, Poland (Table 1). The patients had smoked a minimum of 10 cigarettes per day for at least 5 years preceding the study and ceased smoking for three months of the experiment. The study material, venous blood from the basilic vein, was taken fasting from the study group members at 4 time points: prior to smoking cessation, as well as after the 1st, 2nd, and 3rd months of tobacco abstinence. The patients from the study group ceased smoking and received incentive payments in their workplace for maintaining tobacco abstinence. The patients were also monitored for nonsmoking by their managers and coworkers. Apart from the workplace, the monitoring was conducted at homes by family members interested in smoking cessation by their relatives.

The study also included two control groups composed of 33 subjects.

*Control I*. This group was composed of healthy volunteers (without COPD), nonsmoking either at the time of the study or ever before, not exposed to smoke at home/work, and aged 19 to 73 (mean age = 44.8 ± 15.2 years).

*Control II*. This group was composed of cigarette smokers (smoking at least 10 cigarettes per day for a minimum of the preceding 5 years) with COPD, who did not cease smoking during the experiment, aged 29 to 73 (mean age = 47.7 ± 13.6 years).

Subjects with health problems characterized by a proven disturbance in the activity of lysosomal enzymes were excluded from the study. None of the study subjects were diagnosed with innate AAT deficiency. The research had the approval of the Bioethics Committee at the Collegium Medicum in Bydgoszcz of the Nicolaus Copernicus University in Toruń (nr KB/131/2007) and all subjects had given their written informed consent.

Blood sampling in the nonsmokers (control I) was performed once, while, in the case of the COPD patients who did not cease smoking (control II), blood samples were obtained 4 times at 1-month intervals. As in the case of the study group, blood was taken fasting from the basilic vein.

The patients from the study group and control II were assigned GOLD category A of COPD with low acuteness of the disease and mild symptoms. In 31 patients from the study group, a mild degree of obstruction was determined (forced expiratory volume in 1 second – FEV_1_ > 80% normal value; the FEV_1_/FVC ratio below the lower limit of normal, where FVC is the forced vital capacity of the lungs), while, in 39 subjects, the degree was moderate (50% normal value < FEV_1_ < 80% normal value; the FEV_1_/FVC ratio below the lower limit of normal). Among the patients from control II, 16 of them presented a mild degree of obstruction development, while, in 17 of them, the degree was moderate (the FEV_1_/FVC ratio below the lower limit of normal in all cases). The patients from both groups were not receiving any regular pharmacological treatment. Sporadically, depending on the needs, antihistamines, inhaled steroids, or *β*-mimetics, as well as mucolytic medications, were introduced.

Venous blood samples for testing were collected into dry tubes in order to obtain blood serum. After coagulation, the blood was centrifuged for 10 min at 12,000 g. Until the tests, the obtained blood serum was stored at –20°C. In the blood serum, the activity of the following lysosomal enzymes was determined: AcP, CTS D, and ASA, as well as the activity of the protease inhibitor AAT. The diagnosis of COPD was based on an interview, physical examination, and spirometry (using the KoKo Legend spirometer by Ferraris Systems), whose aim was to confirm the obstructive nature of the disorder.

### 2.1. Assay of *α*_1_-Antitrypsin Activity in Blood Serum

The activity of AAT was determined using the Eriksson method and expressed in mg of trypsin/mL serum [15, 16]. This procedure relies on the evaluation of the level of trypsin inhibited by AAT present in 1 mL of blood serum.

### 2.2. Assay of Lysosomal Enzymes Activity in Blood Serum

The CTS D activity was determined using Anson's method [17]. The substrate was 2% denatured bovine haemoglobin diluted in 100 mL 0.1 M citric phosphate buffer at pH 3.8. The activity of the enzyme was shown by the amount of tyrosine released during enzymatic hydrolysis of the substrate. The AcP activity was determined using Bessey's method [18]. The measure of activity was the quantity of p-nitrophenol generated during the enzymatic hydrolysis of 4-nitrophenylphosphate disodium salt used as a substrate. The activity of ASA was assayed according to Roy's method modified by Błeszyński and Działoszyński [19]. The substrate employed in this case was 4-nitrocatechol sulphate (4-NCS), and the measure recorded was the quantity of 4-nitrocatechol released during enzymatic hydrolysis. The activity of CTS D, AcP, and ASA was expressed in nM/mg of protein/min.

### 2.3. Statistical Analysis

Statistical analysis was conducted using the ANOVA test with post hoc analysis (Tukey's range test) (*STATISTICA v.* 9.1). A hypothesis of the equality of two means was tested. The conformity to the normal distribution was determined on the basis of the Shapiro-Wilk test. The equality of variances was assessed using Levene's test. Differences at a significance level *P* < 0.05 were assumed as statistically significant. Dependencies between the analysed parameters were assessed using correlation matrices. A statistical hypothesis of the significance of the correlation coefficients (*r*) was tested.

## 3. Results

The AAT activity was significantly higher in the blood serum of the patients with COPD from both study group and control II at all time points, as compared with the activity of this protease inhibitor in the healthy subjects from control I (Table 2). The AAT activity in the blood serum of the patients before smoking cessation and the patients from control II before the start of the experiment was higher by approximately 80% (*P* < 0.001) than in the healthy subjects from control I.

**Figure 1:**
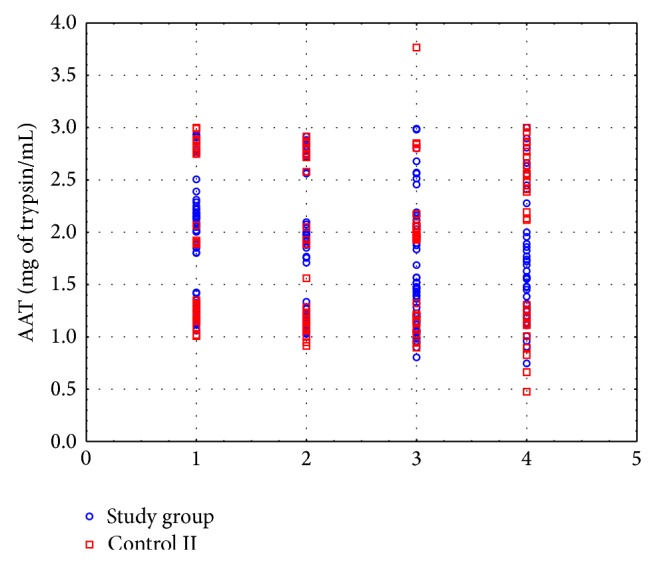
Activity of *α*_1_-antitrypsin (AAT) in the blood serum of each COPD patient who ceased smoking (study group) and of COPD patients who did not cease smoking (control II) at the consecutive study visits. 1: before smoking cessation/at the start of the experiment. 2: after the 1st month of tobacco abstinence/after the 1st month of the study. 3: after the 2nd month of tobacco abstinence/after the 2nd month of the study. 4: after the 3rd month of tobacco abstinence/after the 3rd month of the study.

Tobacco abstinence did not induce any statistically significant changes in the AAT activity. After the 2nd and 3rd months of tobacco abstinence, the AAT activity was 13% lower (*P* > 0.05) and 11% lower (*P* > 0.05), respectively, as compared to the value obtained before smoking cessation. Similarly, no statistically significant changes in the AAT activity were found during the experiment in the patients who did not cease smoking. The AAT activity in the blood serum of the control II subjects at each time point did not differ also in comparison to the activity measured in patients who had ceased smoking (Figure 1).

Neither of the significant differences was found in the activity of the assayed lysosomal enzymes in the blood serum of the patients from both groups and the healthy subjects from control I (Table 2). Tobacco abstinence did not affect significantly the activity of AcP, ASA, and CTS D in the blood serum of the patients with COPD. Likewise, in the subjects from control II, no changes in the activity of the assayed lysosomal hydrolases were observed during the experiment.

**Figure 2:**
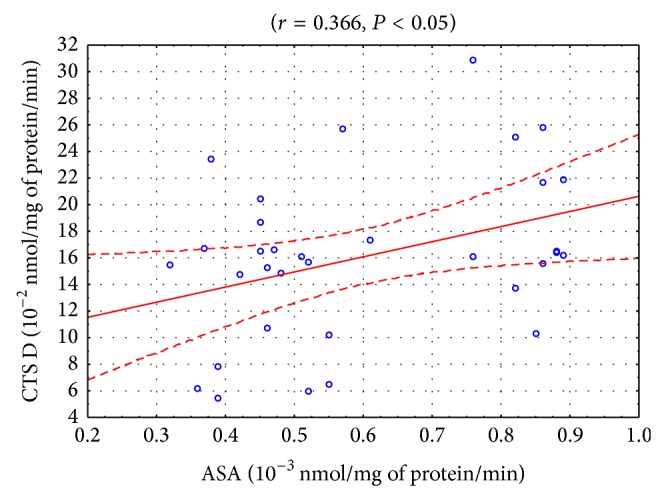
Linear regression (*r* = 0.366, *P* < 0.05) of cathepsin D (CTS D) activity versus arylsulfatase (ASA) activity in the blood serum of COPD patients who did not cease smoking (control II) at the start of the experiment.

**Figure 3:**
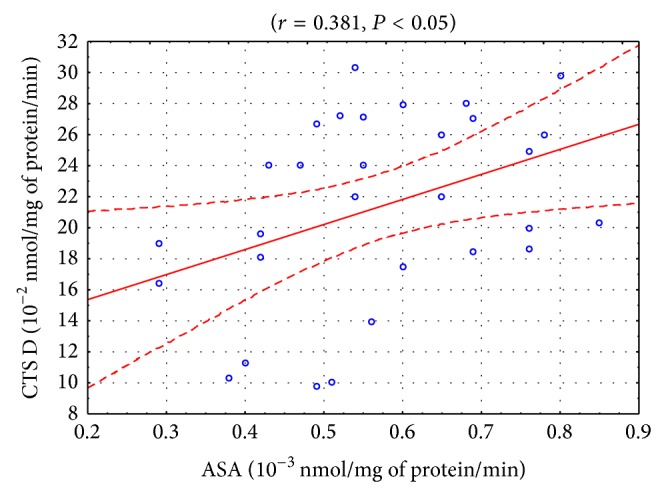
Linear regression (*r* = 0.381, *P* < 0.05) of cathepsin D (CTS D) activity versus arylsulfatase (ASA) activity in the blood serum of COPD patients who did not cease smoking (control II) after the 1st month of the study.

Statistically significant positive correlations were found between the activities of CTS D and ASA in the blood serum of the patients from control II prior to the start of the experiment (*r* = 0.366, *P* < 0.05; Figure 2) and after one month from the start of the experiment (*r* = 0.381, *P* < 0.05; Figure 3). A positive correlation was also observed between the activities of CTS D and AcP in the blood serum of the healthy subjects (*r* = 0.376, *P* < 0.05). Positive correlations between the activities of CTS D and AAT were demonstrated in the patients from the study group after the 1st month of tobacco abstinence (*r* = 0.312, *P* < 0.05) and in the patients from control II after the 1st (*r* = 0.471, *P* < 0.05) and the 2nd months from the start of the experiment (*r* = 0.470, *P* < 0.05). In turn, a negative correlation between these parameters was observed in the blood serum of the patients from control II after the 3rd month from the start of the experiment (*r* = −0.372, *P* < 0.05). A positive correlation was found between the activities of AAT and ASA in the patients from the study group after the 1st month from smoking cessation (*r* = 0.260, *P* < 0.05).

## 4. Discussion

In the patients from either the study group or control II, the activity of AAT in blood serum was statistically significantly higher than in the healthy nonsmoking subjects, which indicates an increased synthesis of the protein in the liver of COPD patients. From the circulation, AAT can enter the lungs and, in addition to locally synthesized AAT, participate in the inhibition of the proteolytic enzymes released in the lungs [20]. Therefore, the higher activity of AAT demonstrates a certain disturbance in the protease-antiprotease balance and its favourable bias toward the increased activity of antiproteolytic defence mechanisms. On the other hand, the higher activity of AAT in blood serum proves the existence of an inflammatory process [21], the root cause of the COPD pathogenesis [6].

Serapinas et al. [20] demonstrated that the increase in the AAT concentration in blood serum is related to smoking, as they observed a higher concentration of this enzyme in current smokers and exsmokers than in never-smokers. Higher level of AAT in the blood serum of smokers was also demonstrated by Linja-Aho et al. [10]. The concentration of AAT was higher in smokers without COPD and in smokers with COPD than in healthy nonsmokers. Smoking cessation for a period of two years in the subjects both with and without COPD resulted in a reduction in the AAT concentration in blood plasma. In this study, no statistically significant changes in the AAT activity after smoking cessation were found in the blood serum of patients with COPD. Probably the time that passed after smoking cessation was too short to affect the observed AAT activity.

It has been proved that an acute increase in the concentration of AAT in blood serum particularly accompanies COPD exacerbations [22]. In turn, Chen et al. [23] observed lower AAT concentration in tobacco smokers with COPD than in smokers without obstructive conditions of the bronchi. It appears that the divergent results presented in the literature may be primarily due to different stage of advancement of inflammatory processes.

In this study, the activity of the lysosomal enzymes AcP, ASA, and CTS D did not differ significantly in a comparison between healthy subjects and patients with COPD. Similarly, smoking cessation for 3 months did not result in statistically significant changes in the activity of the assayed lysosomal hydrolases.

Small amounts of lysosomal enzymes constantly leak from lysosomes into extracellular space and then into the blood. Increased release of lysosomal enzymes is usually related to a general inflammatory process [24]. COPD is associated with local and systemic inflammation [25]. The nonselective nature of lysosomal enzyme leakage is indicated in this study by the statistically significant positive correlations between the activity of CTS D and ASA (Figures 2-3). The lysosomal damage may occur, for example, as a result of oxidative stress which was proved to occur in COPD [6, 7]. On the other hand, the low correlation may indicate a selective penetration of the enzymes as a result of their degranulation and release from cellular lysosomes. Such action is displayed by, for example, IL-8, an inflammation mediator in COPD [26]. Probably, the lack of statistically significant differences in the activity of AcP, ASA, and CTS D may be due to the stage of advancement of COPD (GOLD category A) or may be related to the potential increase in the levels of the inhibitors of those enzymes.

The limited data that occur regard only studies in animals. For example, increased expression of CTS D was detected in the lungs of mice exposed to cigarette smoke [27]. The increase in the activity of acid phosphatase isoforms was demonstrated in the liver and sublingual gland of rats after 25 days of exposure to tobacco smoke [28]. The destabilizing activity of nicotine on lysosomal membranes was also proved by Moździerz et al. [29]. The authors demonstrated an increase in the activity of acid phosphatase and cathepsins D and L in liver and kidney homogenates of mice treated with nicotine (via intraperitoneal injection).

In this study, no changes in the activities of the assayed lysosomal enzymes were demonstrated; however, studies by other authors indicate changes in the activities of these enzymes in tissues treated with nicotine. Therefore, it seems interesting to continue the studies in order to fully understand the potential role of these enzymes in the systemic changes accompanying COPD.

## 5. Conclusions

The obtained results confirm that COPD involves increased AAT activity and unchanged activities of AcP, ASA, and CTS D. Three-month tobacco abstinence does not affect these parameters in peripheral blood. Determining the AAT levels in blood serum can be used in the diagnostics of COPD.

## Figures and Tables

**Table 1:** Patient characteristics.

	Nonsmokers —control I	COPD patients —control II	COPD patients —study group
Number of subjects	33	33	70
Age (years)	44.8 ± 15.2	47.7 ± 13.6	48.8 ± 12.1
Sex (F/M)	15/18	14/19	32/38
Smoking period (years)	—	31.4 ± 10.2	30.9 ± 13.5
Number of packs/year	—	292.0 ± 65.6	287.4 ± 78.3
FEV_1_ (% predicted value)	97.9 ± 13.9	72.9 ± 19.3	73.1 ± 17.5
FVC (% predicted value)	109.5 ± 13.5	94.1 ± 15.8	94.3 ± 18.2
FEV_1_/FVC (%)	84.9 ± 5.8	62.0 ± 7.1	61.5 ± 7.4

FEV_1_: forced expiratory volume in 1 second; FVC: forced vital capacity; FEV_1_/FVC: forced expiratory volume in 1 second/forced vital capacity ratio.

Data expressed as mean $\bar{x}$  ± SD.

**Table 2:** Activity of lysosomal enzymes and *α*_1_-antitrypsin in the COPD patients who ceased smoking and in the representatives of the control groups: COPD patients who did not cease smoking and nonsmokers.

Group	Parameters			
	AcP (10^−2^ nmol/mg of protein/min)	ASA (10^−3^ nmol/mg of protein/min)	CTS D (10^−2^ nmol/mg of protein/min)	AAT (mg of trypsin/mL)
Control I (healthy nonsmokers)	1.45 ± 0.42	0.54 ± 0.13	1.65 ± 0.67	1.01 ± 0.17
COPD patients who did not cease smoking (control II)				
At the start of the experiment	1.57 ± 0.66	0.6 ± 0.2	1.61 ± 0.62	1.82 ± 0.75^**^
After the 1st month of the study	1.65 ± 0.75	0.57 ± 0.15	2.13 ± 0.61	1.83 ± 0.8^**^
After the 2nd month of the study	1.79 ± 0.63	0.6 ± 0.17	1.93 ± 0.6	1.84 ± 0.68^**^
After the 3rd month of the study	1.62 ± 0.47	0.59 ± 0.21	2.05 ± 1.0	1.88 ± 0.82^**^
COPD patients who ceased smoking (study group)				
Before smoking cessation	1.53 ± 0.66	0.57 ± 0.16	1.81 ± 0.78	1.84 ± 0.54^**^
After the 1st month of tobacco abstinence	1.53 ± 0.71	0.55 ± 0.16	2.12 ± 0.56	1.84 ± 0.69^**^
After the 2nd month of tobacco abstinence	1.89 ± 0.71	0.54 ± 0.19	1.97 ± 0.49	1.6 ± 0.59^*^
After the 3rd month of tobacco abstinence	1.6 ± 0.6	0.59 ± 0.21	2.09 ± 0.88	1.64 ± 0.58^*^

AcP: acid phosphatase; ASA: arylsulfatase; CTS D: cathepsin D; AAT: *α*_1_-antitrypsin.

Data expressed as mean $\bar{x}$  ± SD.

Statistically significant differences: versus control I: ^*^ *P* < 0.01, ^**^ *P* < 0.001.
